# Identification and fungicides sensitivity evaluation of the causal agent of cobweb disease on *Lyophyllum decastes* in China

**DOI:** 10.1186/s12866-024-03326-0

**Published:** 2024-05-24

**Authors:** Keqin Peng, Meiling Lin, Xiaoxiao Yuan, Changtian Li, Xiangyu Zeng, Fenghua Tian, Yu Li

**Affiliations:** 1 Department of Plant Pathology, College of Agriculture, Guizhou University, 550025 Huaxi, Guiyang, Guizhou China; 2 Engineering Research Center of Chinese Ministry of Education for Edible and Medicinal Fungi, Jilin Agricultural University, 130118 Nanguan, Changchun, Jilin China; 3 Institute of Edible Mushroom, Guizhou University, 550025 Huaxi, Guiyang, Guizhou China; 4 Guizhou Key Laboratory of Edible Mushroom Breeding, 550025 Guiyang, Huaxi, Guizhou China

**Keywords:** Koch’s postulates, Antibacterial effect, *Hypomyces Odoratus*, Multi-gene phylogeney

## Abstract

**Background:**

Cobweb disease is a fungal disease that commonly affects the cultivation and production of edible mushrooms, leading to serious yield and economic losses. It is considered a major fungal disease in the realm of edible mushrooms. The symptoms of cobweb disease were found during the cultivation of *Lyophyllum decastes*. This study aimed to identify the causative pathogen of cobweb disease and evaluate effective fungicides, providing valuable insights for field control and management of *L. decastes* cobweb disease.

**Results:**

The causal agent of cobweb disease was isolated from samples infected and identified as *Cladobotryum mycophilum* based on morphological and cultural characteristics, as well as multi-locus phylogeny analysis (ITS, RPB1, RPB2, and TEF1-α). Pathogenicity tests further confirmed *C. mycophilum* as the responsible pathogen for this condition. Among the selected fungicides, Prochloraz-manganese chloride complex, Trifloxystrobin, tebuconazole, and Difenoconazole exhibited significant inhibitory effects on the pathogen’s mycelium, with EC50 values of 0.076 µg/mL, 0.173 µg/mL, and 0.364 µg/mL, respectively. These fungicides can serve as references for future field control of cobweb disease in *L. decastes*.

**Conclusion:**

This study is the first report of *C. mycophilum* as the causing agent of cobweb disease in *L. decastes* in China. Notably, Prochloraz-manganese chloride complex demonstrated the strongest inhibitory efficacy against *C. mycophilum.*

**Supplementary Information:**

The online version contains supplementary material available at 10.1186/s12866-024-03326-0.

## Background

**Fig. 1:**
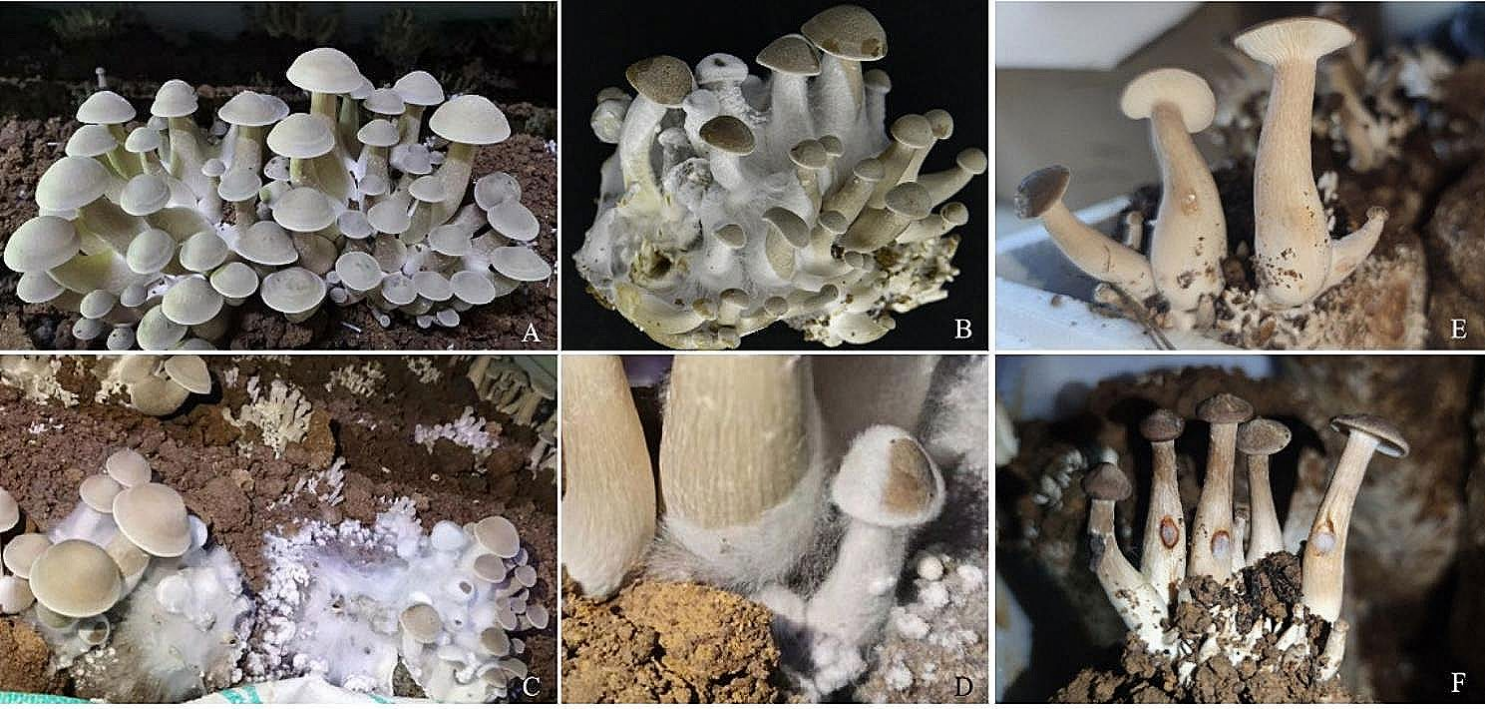
Wild symptoms of causing cobweb disease on *L. decastes* and pathogenicity tests of *C. mycophilum* (2021062102-1). **(A)**, Healthy fruiting bodies of *L. decastes.*** (B-C)**, Rotten fruiting bodies at late stage of the disease. **(D)**, White anamorph spread over *L. decastes*. **(E)**, Pathogenicity tests, 2 days after inoculation, control, asymptomatic. **(F)**, Pathogenicity tests, 2 days after inoculation of *C. mycophilum* 2021062102-1, infected

*Lyophyllum decastes* (Fr.) Singer, commonly known as antler mushroom, is a precious edible and medicinal mushroom (Fig. 1A). It belongs to Lyophyllaceae, Agaricales, Agaricomycetes, Basidiomycota [1]. In China, *L. decastes* is primarily found in Liaoning, Jilin, Heilongjiang, Jiangsu, Qinghai, Sichuan, Guizhou, Yunnan, and Xinjiang provinces [2]. The fruiting bodies of this mushroom are delicate and highly nutritious, with *L. decastes* being renowned for the high content of protein, essential amino acids and polysaccharides (LDS), which exhibit various medicinal properties [3, 4]. According to data from the China Edible Mushroom Association, the production of *L. decastes* in China has experienced rapid growth since 2015, reaching 21,600 tons in 2019 [3]. Currently, cobweb disease is prevalent among edible mushrooms, with its characteristic symptom being the formation of web-like mycelia that initially cover the surface of the fruiting bodies. Over time, the mycelia progressively spread, eventually enveloping the entire fruiting body, leading to wilting and decay of the affected mushrooms [5, 6]. During the late stages of infection, a substantial quantity of conidia is generated, facilitating transmission and the onset of an epidemic, particularly in environments with air circulation. These conidia spread to adjacent fruiting bodies, resulting in secondary infections. Cobweb disease, brown spot disease (caused by *Verticillium* spp.), green mold disease (*Trichoderma* spp.) and brown rot disease (caused by *Hypomyces perniciosus*) are widely recognized as the four most severe fungal diseases affecting numerous mushroom varieties, including *Flammulina velutipes*, *Pleurotus eryngii* var. *tuoliensis*, *Lentinula edodes*, *P. eryngii*, *Agaricus bisporus*, *Coprinus comatus*, *Ganoderma lingzhi* [5, 7–11], These diseases pose significant challenges to the mushroom industry in China, exerting a considerable impact on the agricultural economy and the income of mushroom farmers [8–11].

Cobweb disease on mushrooms also occurs in other regions and countries. Instances of cobweb disease caused by *C. mycophilum* and *C. varium* have been documented on *A. bisporus*, *P. eryngii*, and *F. velutipes* in Korea [12]. In the same period, *C. mycophilum* also caused cobweb disease on *A. bisporus* at Castilla La Mancha, Spain [13, 14]. This fungal infection is increasingly becoming a serious concern in South Africa’s edible mushroom cultivation. The cobweb disease is mostly caused by *Cladobotryum* spp., and the same pathogen can cause disease on different edible mushrooms. For example, *C. mycophilum* has been observed to induce cobweb disease in *P. eryngii*, *P. ostreatus*, and *G. lingzhi* [14–16]. Moreover, multiple pathogens can contribute to diseases in the same edible mushroom. For instance, both *C. varium* and *C. asterophorum* can cause cobweb disease in *Hymenopellis raphanipes*, while *C. mycophilum* and *C. dendroides* can lead to cobweb disease in *L. edodes* and *A. bisporus* [12, 17–21]. Furthermore, *C. varium* has been linked to cobweb disease in *F. velutipes* [12]; *C. protrusum* in *C. comatus* [22], *C. cubitense* in *A. cornea* [10, 23], and *C. asterophorum* in *H. raphanipes* [18]. Cobweb disease is caused by a wide variety of pathogens and poses a threat to multiple edible mushroom species. Therefore, it is urgent to conduct research on the prevention and control of cobweb disease. To prevent disease outbreaks, attention should be paid to improving the disease resistance of mushroom varieties. In order to prevent disease outbreaks, it is crucial to focus on enhancing the disease resistance of mushroom varieties. Additionally, attention should be given to ensuring the vitality and purity of spawn, maintaining cleanliness in culture rooms, implementing soil disinfection measures, and following standard operating procedures throughout the cultivation process. However, the cobweb disease is common in mushrooms [24], and there is no simple and effective means to control cobweb disease. To tackle disease occurrence, fungicides are often applied as preventive treatments for the extensive outbreaks [25].

In June 2021, the authors conducted a disease investigation in the cultivation area of *L. decastes* in Baiyun District, Guiyang, Guizhou, China, and discovered a prevalent disease suspected to be cobweb disease. The normal growth temperature for *L. decastes* fruiting bodies is typically 15–18 ℃, with humidity levels ranging from 90 to 98%. However, due to the high outdoor temperatures experienced in the days preceding and following the investigation, the temperature inside the mushroom house rose to over 22 ℃. This, combined with the high humidity in the mushroom room, resulted in the outbreak of diseases. The disease exhibited a high level of infectivity and severity, with the incidence rate reaching 3–5% within just a few days. This poses a significant threat to *L. decastes* cultivation and production. The isolated pathogen was identified through a combination of morphological characteristics and phylogenetic analysis, and its pathogenicity was confirmed using Koch’s postulates. Additionally, the antifungal effects of nine fungicides were analyzed using the mycelial growth rate method on the pathogen. As a result, the findings of this study will serve as a valuable reference for comprehensive disease prevention and control measures in *L. decastes* cultivation.

## Results

### Disease symptoms identification

The symptoms of cobweb disease were obvious in the middle and late growth stage of cultivation and tended to be aggravated with the increase of the number of fruiting boides. The pathogen initially appeared at the base of the stalk or on the surface of the covering soil or fruit bodies. Initially, the roots of the fruiting bodies were covered with white, coarse, and cobweb-like mycelia, which then spread along the stalk to the cap. Subsequently, the white, fluffy mycelia of the pathogen rapidly spread, covering the surrounding soil and fruiting bodies. Eventually, the fruiting bodies rotted and shrank, turning dark brown and emitting a rancid odor. They were covered with a mass of conidia, which could rapidly spread to adjacent fruiting bodies (Fig. 1B-D). The disease spread rapidly throughout the entire cultivation shed, leading to abnormal mushroom growth and failed harvest.

### Pathogenicity results

A total of 12 isolates were obtained from the infected fruiting bodies, among which strains 2021062102-1 (GUCC202106) and 2021062102-3 (GUCC202107) exhibited pathogenicity. Pathogenicity tests revealed that symptoms of cobweb disease became visible 2 days after inoculation (Fig. 1F). Hair-like filaments were produced at the inoculation site and gradually spread, resulting in distinct symptoms observed 2 days post-inoculation. These symptoms observed through artificial inoculation were similar to those observed in the field. The control group showed no symptoms (Fig. 1E). The pathogens were consistently re-isolated from the infected fruiting bodies of *L. decastes*, confirming Koch’s postulates, and their morphological characteristics matched those of the initially inoculated strains.

### Morphological description

**Fig. 2:**
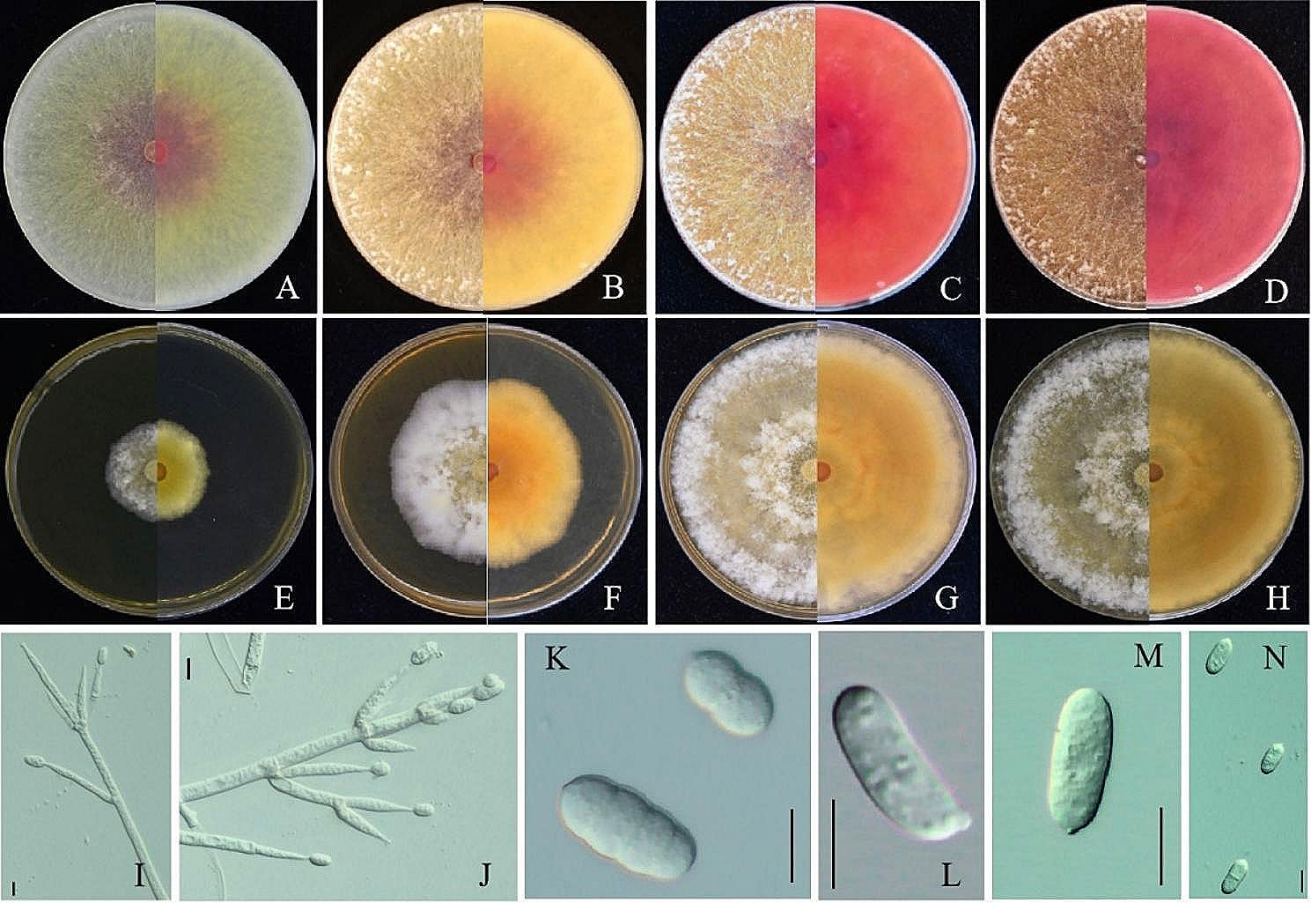
Morphology characterization of *C. mycophilum* (2021062102-1). **(A-D)**, Colony morphology on PDA medium at 25℃. **A**: after 3 days; **B**: after 5 days; **C**: after 10 days; **D**: after 14 days. **(E-H)**, Colony morphology on MEA medium at 25℃. **E**: after 3 days; **F**: after 5 days; **G**: after 10 days; **H**: after 14 days. **(I-J)**, Conidiophores cells straight, hyaline, branching profuse, tips simple, Bar = 10 μm. **(K-N)**, Conidia, with 0–3 septa, Bar = 10 μm

Colonies grew rapidly on a 90 mm PDA plate, covering the entire petri dish after 3 days at 25℃. The reverse side initially appeared yellowish ochraceous, but turned roseous or brownish red within 10 days (Fig. 2A-D). The aerial mycelia of the colony were lush and cotton-like, with an abundance of conidia. On a 90 mm MEA medium, the colonies grew slowly and covered the entire culture dish after 10 days at 25℃, producing a large number of dense conidia (Fig. 2E-H). The shape and size of the conidia were recorded and measured using a compound light microscope (Zeiss Scope 5 with camera AxioCam 208 color). The conidiophores are straight, hyaline, with profuse branching, and simple tips, measuring 24.5–37.6 × 4.0–6.7 μm (*n* = 30) (Fig. 2I-J). The conidia were mostly ellipsoidal, hyaline, with 0–3 septa, rounded bases, and slight constriction at the septum, measuring 17.3–27.2 × 7.9–10.4 μm (*n* = 50) (Fig. 2K-N). Based on the morphological characteristics, the isolate 2021062102-1 was identified as *C. mycophilum.*

### Phylogenetic analyses

**Fig. 3:**
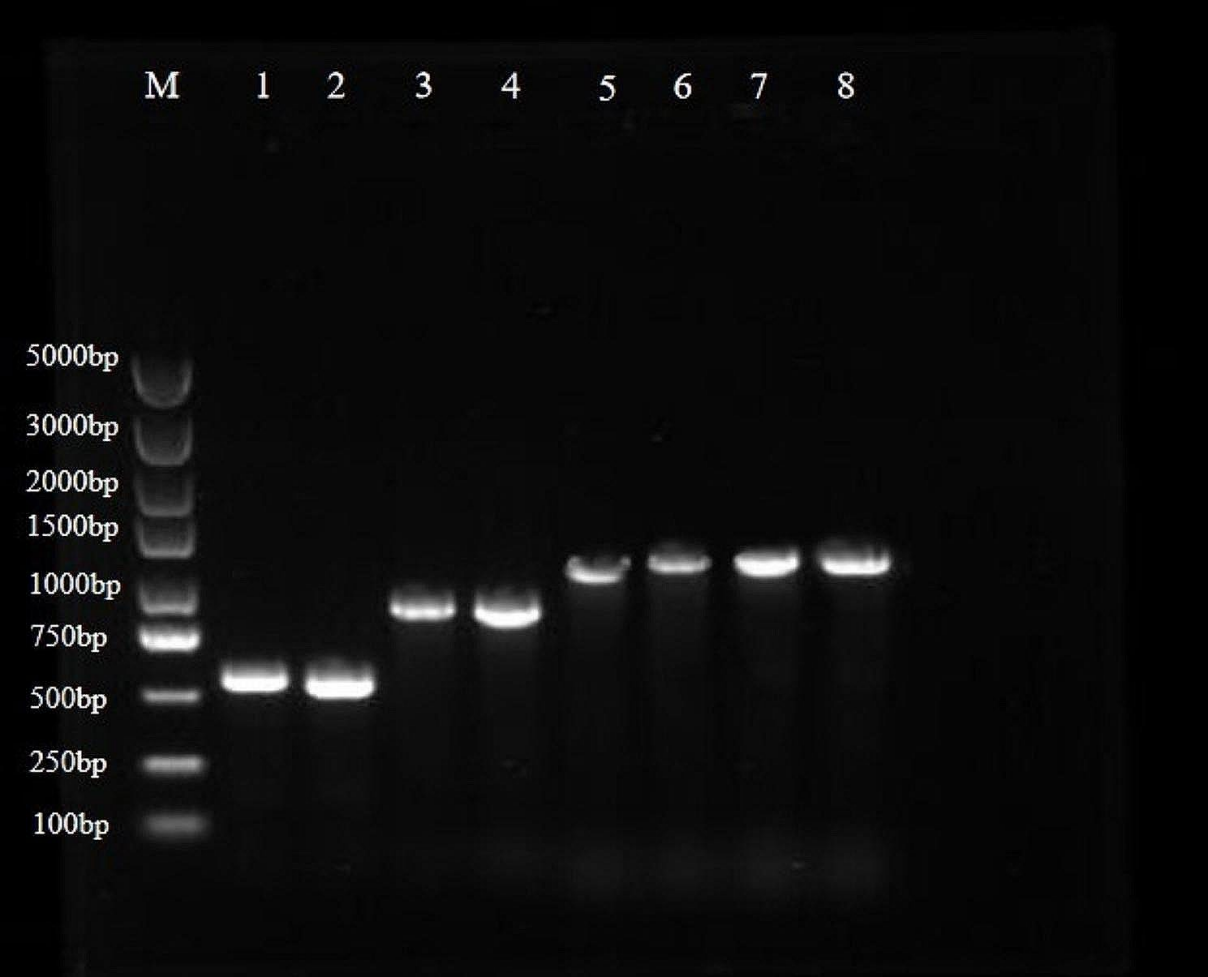
The amplification images of all genes ( ITS, TEF1-α, RPB1 and RPB2 ). Lane M indicates the marker; Lanes 1–2 represent the amplified ITS gene, about 650 bp; Lanes 3–4 represent the amplified TEF1-α gene, about 900 bp; Lanes 5–6 represent the amplified RPB1 gene, about 1100 bp; Lanes 7–8 represent the amplified RPB2 gene, about 1100 bp

**Fig. 4:**
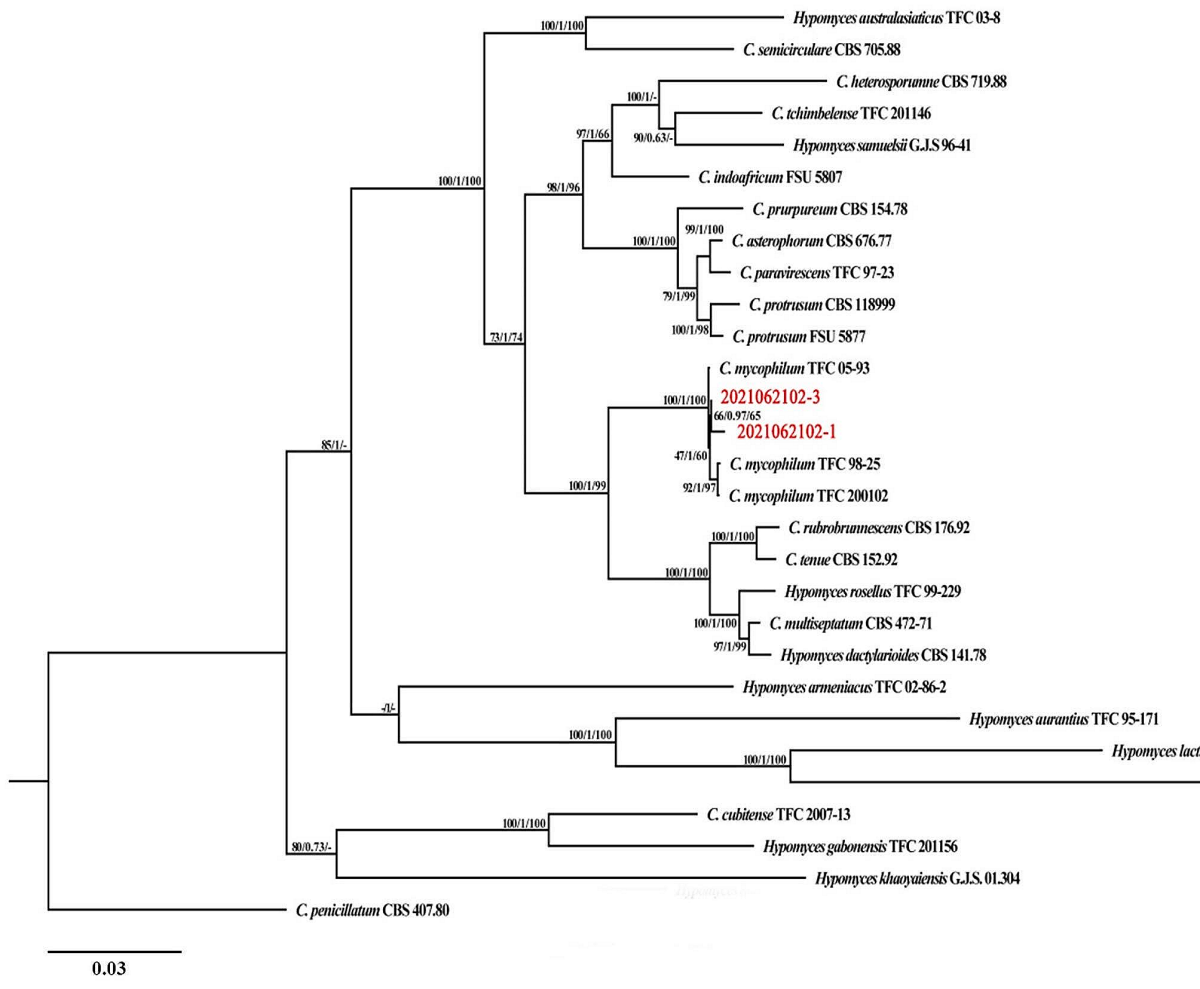
Multi-gene phylogenetic tree based on combined ITS, RPB1, RPB2, TEF1-α sequences. ML and MP bootstrap values greater than 50% are reported above the branches, BI values > 0.90 are shown next to topological nodes and separated by “/”. Bootstrap values < 50% and BI values < 0.90 are labeled with “-”. The tree was rooted to *Cladobotryum penicillatum* CBS 407.80

The ITS-rDNA, RPB1, RPB2, and TEF1-α genes of the two isolates (GUCC202106: 2021062102-1, GUCC202107: 2021062102-3) were amplified and sequenced using primers ITS4/ITS5, cRPB1Af/RPB1Cr, RPB2-5f/RPB2-7cR, and EF1-983f/EF1-2218r, respectively (Fig. 3). A multigene phylogenetic tree, generated using the maximum-likelihood method based on the concatenated ITS-RPB1-RPB2-TEF1-α sequences, confirmed that the multiple isolates belonged to *C. mycophilum*. According to comprehensive identification of the phylogenetic analysis, morphological characteristics and cultural characteristics, the isolates were identified as *C. mycophilum* (Fig. 4). The results reproduced the similarity to morphological identification.

### **Effect of different fungicides on the cobweb disease pathogen of*Lyophyllum decastes***

The effect of nine fungicides on the radial growth of the pathogen (2021062102-1) was studied to determine which fungicides are highly effective against the pathogen (Table 1). The average radial growth of the fungus was significantly influenced by different fungicides. The results of the nine fungicides showed good inhibitory effects on the pathogen in PDA medium. Among them, Prochloraz-manganese chloride complex (50% WP) was the most effective in controlling the pathogen, with an EC50 of 0.076 µg/mL. Trifloxystrobin and tebuconazole (75% WDG) were the second most effective, with an EC50 of 0.173 µg/mL. Additionally, Difenoconazole (10% WDG) showed better effectiveness in controlling the pathogen, with an EC50 of 0.364 µg/mL. However, the inhibitory effect of Carvacrol (5% SL) was slightly weaker than that of the other fungicides.

**Table 1:** The virulence effects of nine kinds of fungicides on the pathogen

Fungicides	Treatment concentration µg mL^− 1^					Toxicity regression equation	EC50/(µg mL^− 1^)	Correlation coefficient
	T1	T2	T3	T4	T5			
Carvacrol (5% SL)	500.00	100.00	20.00	4.00	0.80	y = 3.0897x + 2.4323	6.777	0.9711
Osthol (1% EW)	50.00	10.00	2.00	0.40	0.08	y = 1.0215x + 4.3536	4.294	0.9619
Eugenol (0.3% SL)	30.00	6.00	1.20	0.24	0.05	y = 2.0136x + 5.0131	0.985	0.9725
Propiconazole (25% EC)	5.00	2.50	1.25	0.63	0.31	y = 1.6649x + 5.4195	0.560	0.9163
Triadimefon (20% EC)	5.00	2.50	1.25	0.63	0.31	y = 1.0460x + 4.8275	1.462	0.9979
Trifloxystrobin and tebuconazole (75% WDG)	1.39	0.35	0.09	0.02	0.01	y = 1.3942x + 6.0633	0.173	0.9572
Prochloraz-manganese chloride complex (50% WP)	1.00	0.33	0.11	0.04	0.01	y = 0.7724x + 5.8659	0.076	0.9759
Pyraclostrobin (10% WDG)	2.40	1.20	0.60	0.30	0.15	y = 1.4208x + 4.7917	1.402	0.9805
Difenoconazole (10% WDG)	10.00	1.00	0.10	0.01	0.00	y = 0.8038x + 5.3524	0.364	0.9688

## Discussion

Cobweb disease has been reported in mushroom-growing countries worldwide, leading to economic losses and significantly hindering the development of the edible mushroom industry. In the mid-1990s [26, 27], cobweb disease was initially discovered on *A. bisporus* and subsequently on *P. eryngii*, *C. comatus*, and *P. ostreatus* [22, 28–31]. As a rare new cultivated variety of edible fungus in China, *L. decastes* has not been spared and has been affected by cobweb disease. Most cases of cobweb disease are caused by *Cladobotryum* spp., the sexual type of *Hypomyces aurantius* is also capable of causing cobweb disease on *Hypsizygus marmoreus* [6]. The causal agents of cobweb disease vary for different edible mushrooms in different regions [11]. Cobweb disease caused by *C. mycophilum* affects a wide range of edible mushrooms including *P. eryngii* [32], *A. bisporus* [8, 13, 14], *P. ostreatus* [14, 29], *G. lingzhi* [11], *Albatrellus* sp., *Lactarius mitissimus*, *L. mitissimus* cf. *vellereus*, *Russula* sp., *Coniophora* sp., *Megacollybia platyphylla*, *Inocybe* sp., *Armillaria mellea*, and *Lycoperdon pyriforme* [12, 29, 33, 34]. In this study, it was also identified as the pathogen affecting *L. decastes*. The mycelia of *C. mycophilum* on *L. decastes* exhibit rapid growth and prolific conidia production, resulting in a high rate of reproduction. Furthermore, cobweb disease is a soil-borne fungal disease, and its pathogen can persist in the soil for extended periods, continuously causing infections, which makes control measures challenging.

At present, the control of diseases in edible mushrooms primarily relies on chemical agents. Research has demonstrated that Prochloraz-manganese chloride complex, Carbendazim and Propione have certain inhibitory effects in the prevention and control of cobweb disease, among which Prochloraz-manganese chloride complex has a wide range and good antibacterial effect [32, 35–38]. To achieve better control of the cobweb disease caused by *C. mycophilum* on *L. decastes*, we selected nine fungicides to assess their indoor toxicity against strain 2021062102-1. These included six chemical fungicides and three biological fungicides. The results showed that Prochloraz-manganese chloride complex, Trifloxystrobin and tebuconazole, and Difenoconazole exhibited strong toxicity and a good inhibitory effect on the pathogen of cobweb disease. Eugenol in biological fungicides has a good inhibitory effect on the pathogen of cobweb disease. These agents can provide new insights for selecting low-toxicity and high-efficiency options for field control of cobweb disease. However, while chemical fungicides can effectively control the occurrence of cobweb disease, prolonged usage may have potential harmful effects on the environment and human health. Furthermore, the continuous use of the same chemical fungicides can lead to pathogen resistance, ultimately reducing the overall effectiveness of preventing and controlling the spread of infectious diseases [21, 39]. Therefore, in order to reduce the use pressure of chemical fungicides, this study selected Carvacrol, Osthole and Eugenol three low-toxic, non-polluting biological pesticides for indoor toxicity testing. The results showed that these three fungicides all exhibited good antibacterial effects. Among them, Eugenol had the most prominent inhibitory effect and could alleviate the pressure of chemical fungicides to a certain extent. This finding provides useful reference for exploring more environmentally friendly and safe chemical control methods.

*C. mycophilum* is a fungus that poses a significant threat to the mushroom cultivation industry due to its rapid growth and ability to spread quickly during the fruiting body stage. This can result in substantial economic losses for growers. To combat this, it is essential to implement effective control measures for cobweb disease as early as possible during the cultivation process. Our study has identified fungicides that demonstrate low toxicity and high efficacy in controlling the pathogenic mycelium, offering promising prospects for their use in the field. However, managing the spread of this disease requires a comprehensive approach that encompasses cultural practices, physical and biological preventive measures, and ongoing monitoring to delay the development of resistance and maintain effectiveness. Ultimately, the success of these measures directly impacts the yield and profitability of mushroom cultivation, making careful management of cobweb disease critical for the industry’s future.

To prevent and control the disease, it is essential to prioritize prevention and implement comprehensive measures. The selection of healthy and high-quality edible mushroom strains plays a vital role in the industry’s success. Additionally, maintaining a clean cultivation environment, implementing effective management measures, and closely monitoring for disease detection, treatment, and prevention are necessary steps. After completing the planting process, thorough cleaning and disinfection of the mushroom house are crucial to prevent pathogen residues from affecting future cultivation. In addition, chemical control and biological control should be combined with safe and healthy agricultural control to form a healthy and comprehensive prevention. Implementing safe and efficient prevention and control methods is the most effective way to minimize economic losses.

## Conclusions

In this study, the pathogen responsible for *L. decastes* cobweb disease was isolated and identified as *C. mycophilum* through pathogenicity tests, morphological analysis, and multi-locus phylogeny analysis (ITS, RPB1, RPB2 and TEF1-α). This marks the first report of *C. mycophilum* causing cobweb disease on *L. decastes* in China. Additionally, the indoor toxicity of fungicides was determined using the mycelial growth rate method. Among the nine fungicides tested, Prochloraz-manganese chloride complex (50% WP) had the best inhibitory effect, with an EC50 value of 0.076 µg/mL. Trifloxystrobin and tebuconazole (75% WDG), as well as Difenoconazole (10% WDG), followed with EC50 values of 0.173 µg/mL and 0.364 µg/mL, respectively. These findings provide important insights for field control of *L. decastes*.

## Methods

### Pathogen isolation

Three infected fruiting bodies of *L. decastes* were collected from a mushroom cultivation base, Guiyang (106°43′25″ N, 26°43′41″ E), Guizhou Province, China, on June 22nd 2021. Each infected fruiting body was cleaned with flowing water and disinfected on the surface. Secondly, sections with about 0.3 cm square from the infected fruiting body was cut off and surface sterilized with the following steps: immersed in 95% ethanol for 1 min, washed with ddH_2_O 2 times, immersed in 75% ethanol for 30 s, and suspensions were spread on a potato dextrose agar (PDA) plate with three duplicates and incubated at 25℃ in dark. The pathogen of each duplicate was re-isolated and purified while the single colonies formed [40]. All cultures were deposited to Culture Collection of the Department of Plant Pathology, College of Agriculture, Guizhou University, China (GUCC).

### Pathogenicity tests

All isolates were tested for pathogenicity using 2–3 cm high fruiting bodies, following a modified protocol based on Tian et al.‘s study [41]. Ten healthy fruiting bodies were inoculated, with sterilized distilled water serving as the control. All treated fruiting bodies were maintained in the same mushroom-growing space, under specific conditions (16–18℃, 90–95% relative humidity). The pathogenicity test was assessed over a period of four days. The *C. mycophilum* strains were then re-isolated from the infected fruiting bodies. All experiments were conducted in triplicate.

### Morphological and molecular characterization

For the morphological observations of the colonies, the strains were grown on PDA and 1.5% malt extract agar (MEA) medium at 25 °C in dark [24]. Colony characteristics and microscopic morphological characteristics of mycelia, conidiophore, and conidia were observed at 3, 5, 10 and 14 days. Conidia were measured from each isolate. The isolates were then identified based on the morphological characteristics of the conidia and conidiophores according to the descriptions from Gams and Hoozemans [42], Rogerson and Samuels [43]. Additionally, the molecular characteristics of the isolates, total genomic DNA was extracted from the colony of the isolates using a CWBIOTECH Plant Genomic DNA Kit (Changping, Beijing, China) following the manufacturer’s protocol. PCR was set up using the following primers for amplification of different gene regions. The internal transcribed spacer (ITS) region of the rDNA gene cluster was amplified using primers ITS4/ITS5 [44]. Three protein-coding genes were also amplified using the following primers: the partial translation elongation factor 1-α (TEF1-α: EF1-983f/EF1-2218r) [45, 46], RNA polymerase I second largest subunit (RPB1: cRPB1Af/RPB1Cr) [47], and RNA polymerase II second largest subunit (RPB2: fRPB2-5f/fRPB2-7cR) [24, 48].

The PCR was conducted in an Applied Biosystems, ProFlex™ PCR (Thermo Fisher Scientific, Waltham, Massachusetts, USA). The PCR reaction was performed with a 50 µL mixture comprising 3.2 µL of dNTP mix (2.5 mMˑµL^− 1^), 0.2 µL of Taq polymerase (5 U µL^− 1^), 2 µL of genomic DNA (50 ngˑµL^− 1^), 4 µL of polymerase buffers (10 × µL^− 1^, Takara, Japan), and 2 µL of each primer (25 mM µL^− 1^). Amplification of the ITS region was performed as follows: initial denaturation at 94 °C for 5 min, 30 cycles of 30 s at 94 °C, 30 s at 50 °C, 30 s at 72 °C, and with a final extension of 10 min at 72 °C. For amplifying the TEF1-α protein-coding genes programming for an initial denaturation at 94℃ for 3 min followed by 35 cycles of 15 s at 94℃, 15 s at 55℃ and extension at 72℃ for 15 s; and RPB1 region: initial denaturation 5 min at 94 °C, 30 cycles of 30 s at 94 °C, 30 s at 55 °C, 30 s at 72 °C; for RPB2 region: initial denaturation at 95℃ for 3 min followed by 35 cycles of 15 s at 94℃, 15 s at 52℃ and extension at 72℃ for 30 s; and with the same final extension at 72℃ for 10 min. Electrophoresis was performed on 0.8% agarose gels stained with Gel Green (Sangon Biotech (Shanghai) Co.,Ltd.). PCR products were sequenced by the same primers used for amplification by Qingke Biotech (Chengdu) Co., Ltd.

The sequences of ITS, RPB1, RPB2 and TEF1-α genes from representative ex-type strains were selected for phylogenetic analyses and extracted from GenBank using BLAST. The obtained sequences were visualized and aligned using BioEdit [49] and compared against the non-redundant nucleotide collection (nr/nt) sequences present in the NCBI GenBank database using the Basic Local Alignment Search Tool (BLASTn) tool (https://blast.ncbi.nlm.nih.gov/Blast.cgi). As for building the phylogenetic trees, maximum likelihood (ML), maximum parsimony (MP) and Bayesian inference (BI) were performed at the CIPRES web portal [50]. 24 phylogenetically related species of *Cladobotryum*, as *C. asterophorum*, *C. paravirescens*, *C. protrusum*, *C. prurpureum*, *Hypomyces subiculosus*, *H. samuelsii*, *C. tchimbelense*, *C. heterosporumne*, *C. indoafricum*, *C. multiseptatum*, *H. dactylarioides*, *H. rosellus*, *C. rubrobrunnescens*, *C. tenue*, *C. mycophilum*, *C. semicirculare*, *H. australasiaticus*, et al. were used for phylogenetic analysis [24] (Table 2).

**Table 2:** Information of *Cladobotryum spp.* used in this study for phylogenetic analyses

Species	Strain	GenBank accession number			
		ITS	RPB2	TEF-1a	RPB1
*C. asterophorum*	CBS 676.77	FN859395	FN868649	FN868712	FN868776
*C. paravirescens*	TFC 97 − 23	FN859406	FN868660	FN868724	FN868787
*C. protrusum*	CBS 118,999	FN859408	FN868662	FN868726	FN868789
*C. protrusum*	FSU 5877	FN859411	FN868665	FN868729	FN868792
*C. prurpureum*	CBS 154.78	FN859415	FN868669	FN868733	FN868796
*Hypomyces samuelsii*	G.J.S. 96 − 41	FN859448	FN868702	FN868766	-
*C. tchimbelense*	TFC 201,146	FN859419	FN868673	FN868737	FN868800
*C. heterosporumne*	CBS 719.88	FN859398	FN868653	FN868716	FN868780
*C. indoafricum*	FSU 5807	FN859399	FN868654	FN868717	FN868781
*C. multiseptatum*	CBS 472.71	FN859405	FN868659	FN868723	FN868786
*Hypomyces dactylarioides*	CBS 141.78	FN859429	FN868683	FN868748	FN868809
*Hypomyces rosellus*	TFC 99–229	FN859441	FN868695	FN868759	FN868820
*C. rubrobrunnescens*	CBS 176.92	FN859416	FN868670	FN868734	FN868797
*C. tenue*	CBS 152.92	FN859420	FN868674	FN868738	FN868801
*C. mycophilum*	TFC 200,102	FN859433	FN868687	FN868752	FN868813
*C. mycophilum*	TFC 98 − 25	FN85943	FN868688	FN868753	FN868814
*C. mycophilum*	TFC 05–93	FN859436	FN868690	FN868755	FN868816
*C. semicirculare*	CBS 705.88	FN859417	FN868671	FN868735	FN868798
*Hypomyces australasiaticus*	TFC 03–8	FN859428	FN868681	FN868746	FN868807
*Hypomyces khaoyaiensis*	G.J.S. 01-304	FN859431	FN868685	FN868750	-
*Hypomyces armeniacus*	TFC 02–86/2	FN859424	FN868678	FN868742	FN868804
*C. cubitense*	TFC 2007-13	AM779857	FN868652	FN868715	FN868779
*Hypomyces gabonensis*	TFC 201,156	FN859430	FN868684	FN868749	FN868810
*Hypomyces aurantius*	TFC 95–171	FN859425	FN868679	FN868743	FN868805
*Hypomyces lactifluorum*	TAAM 170,476	FN859432	EU710773	FN868751	FN868812
*Hypomyces subiculosus*	TFC 97–166	FN859452	FN868706	FN868770	FN868829
*C. penicillatum*	CBS 407.80	FN859407	FN868661	FN868725	FN868788

### **Screening of fungicides for prevention and control of cobweb disease causal agent on*Lyophyllum decastes***

Referring to the fungicides with high efficacy and low toxicity used for the control of cobweb disease on other edible mushroom, we selected various fungicides, including Carvacrol (5% SL), Osthol (1% EW), Eugenol (0.3% SL), Propiconazole (25% EC), Triadimefon (20% EC), Trifloxystrobin and tebuconazole (75% WDG), Prochloraz-manganese chloride complex (50% WP), Pyraclostrobin (10% WDG) and Difenoconazole (10% WDG). Preliminary indoor screening of fungicides for prevention and control of cobweb disease agent on *L. decastes*: the test method was modified appropriately [51]. According to the active ingredients, nine kinds of low toxic fungicides were diluted with sterile water to make stock solutions of certain concentrations. In order to determine the concentration range of each fungicide, a pre-test was carried out with a concentration gradient of 5 times for each fungicide. According to the volume ratio, the PDA medium containing fungicide was prepared with the amount of storage fluid: PDA = 1:9 in a Petri dish with diameter of 9.0 cm. The pathogen filaments (2021062102-1) which were cultured and grown on PDA medium at 25 °C in dark for 4 days were made into cake with a 5 mm hole punch. PDA medium with equal amount of sterile water without fungicide was used as control. The fungus cakes were transferred into the prepared medium, and incubated at 24 °C in dark for 4 days. In this process, the growth of pathogen was observed to determine the initial concentration of each fungicide. Selecting the fungicide that could inhibit the pathogen and conduct further concentration screening test. According to the pretest results, each fungicide was diluted into 6 concentration gradients according to the effective components. The method of inoculation and culture for each treatment was the same as above. The diameter of colonies was measured with crossing method [18], when colonies in control almost covered the Petri dish. Inhibitory percentage on mycelia growth was calculated after treatment with different concentrations and fungicides. Inhibition of mycelial growth (%) = [(dimeter of mycelium in control -diameter of mycelium in treatment)/dimeter of mycelium in control]x100. Each treatment was repeated three times. The EC_50_ value of each fungicide was evaluated by using ANOVA in three replicates. The ANOVA was performed as per Duncan’s multiple range test to determine the significant differences (* *p* < 0.05) [52].

### Data analysis

All statistical analyses were conducted in MS Excel and SPSS statistics (version 19.0) software. The ANOVA was performed as per Duncan’s multiple range test to determine the significant difference (* *p* < 0.05).

### Electronic supplementary material

Below is the link to the electronic supplementary material.

Supplementary Material 1

## Data Availability

The datasets generated during the current study, which include the sequences of the two isolates identified as GUCC202106 (2021062102-1) and GUCC202107 (2021062102-3), are available in the GenBank repository under the following accession numbers: ITS: OK285275, OK285276; RPB2: OK458561, OK458562; TEF1-α: OK448484, OK448485; RPB1: OK513067, OK513068.
