# Supplementary material for: Identification and fungicides sensitivity evaluation of the causal agent of cobweb disease on Lyophyllum decastes in China
Source: BMC Microbiol. 2024 May 24;24:180. doi: 10.1186/s12866-024-03326-0 (PMC11127302; doi:10.1186/s12866-024-03326-0)
Supplement: Supplementary file 1 — Supplementary Material 1 [file 12866_2024_3326_MOESM1_ESM.docx]

The virulence effects of nine kinds of fungicides on the pathogen are shown in the following figure, and the treatment concentrations T1-T5 are consistent with Table 2.

CK

T5

T4

T3

T2

T1


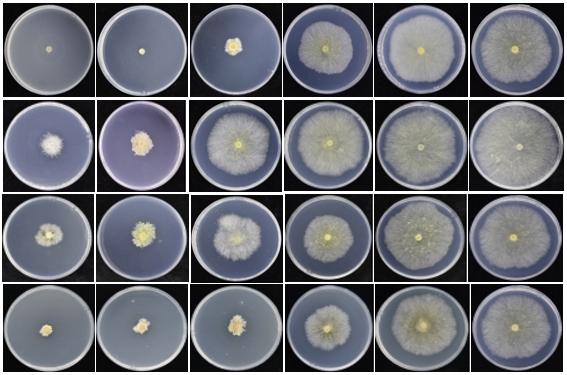

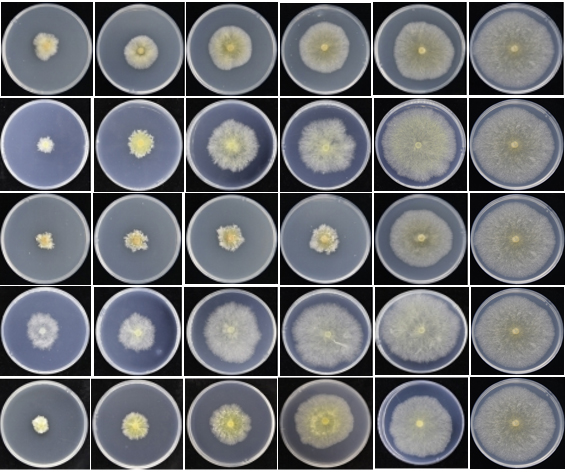


Trifloxystrobin and tebuconazole (75% WDG)

Difenoconazole (10% WDG)

Prochloraz-manganese chloride complex (50% WP)

Pyraclostrobin (10% WDG)

Eugenol (0.3% SL)

Osthol (1% EW)

Carvacrol (5% SL)

Propiconazole (25% EC)

Triadimefon (20% EC)
